# Impact of insulin on primary arcuate neurons culture is dependent on early-postnatal nutritional status and neuronal subpopulation

**DOI:** 10.1371/journal.pone.0193196

**Published:** 2018-02-21

**Authors:** Lyvianne Decourtye, Maud Clemessy, Erik Mire, Tatiana Ledent, Laurence Périn, Iain C. Robinson, Yves Le Bouc, Laurent Kappeler

**Affiliations:** 1 Sorbonne Université, INSERM, Centre de Recherche St-Antoine, CRSA, Paris, France; 2 Sorbonne Université, AP-HP, Hôpital Armand-Trousseau, Paris, France; 3 MRC, National Institute for Medical Research, Division of Molecular Neuroendocrinology, London, United Kingdom; University Paris Diderot, FRANCE

## Abstract

Nutrition plays a critical role in programming and shaping linear growth during early postnatal life through direct action on the development of the neuroendocrine somatotropic (GH/IGF-1) axis. IGF-1 is a key factor in modulating the programming of linear growth during this period. Notably, IGF-1 preferentially stimulates axonal growth of GHRH neurons in the arcuate nucleus of the hypothalamus (Arc), which is crucial for the proliferation of somatotroph progenitors in the pituitary, thus influencing later GH secretory capacity. However, other nutrition-related hormones may also be involved. Among them, insulin shares several structural and functional similarities with IGF-1, as well as downstream signaling effectors. We investigated the role of insulin in the control of Arc axonal growth using an *in vitro* model of arcuate explants culture and a cell-type specific approach (GHRH-eGFP mice) under both physiological conditions (normally fed pups) and those of dietary restriction (underfed pups). Our data suggest that insulin failed to directly control axonal growth of Arc neurons or influence specific IGF-1-mediated effects on GHRH neurons. Insulin may act on neuronal welfare, which appears to be dependent on neuronal sub-populations and is influenced by the nutritional status of pups in which Arc neurons develop.

## Introduction

An essential aspect of development is linear growth. It is under the control of both genetic and environmental factors and is necessary to insure reproductive success during adulthood. Up to 90% of variation in adult height may be linked to genetic factors [1, 2]. However, several studies have shown that only a fraction of the variability in normal height can be associated with polymorphisms by single nucleotide polymorphism-genome wide association studies (SNP-GWAS) [2–4], suggesting that the environment also plays a crucial role. Indeed, increasing evidence suggests that environmental factors during the perinatal period are important in determining life and growth trajectories. The mechanism involved is known as the predictive adaptive response and is thought to underlie the developmental origin of health and diseases (DOHaD) hypothesis.

Among environmental factors, nutritional status during the perinatal period is known to influence growth trajectory programming. We have previously shown that the programming of linear growth in mammals is associated with the nutritional supply during early postnatal life (*i*.*e*. during lactation, P1-P16) [5]. Notably, dietary restriction during lactation is sufficient to strongly alter growth trajectories, leading to long-lasting consequences which persist into adulthood. Such programming of linear growth involves stimulation of the neuro-endocrine somatotropic (GH/IGF-1, Growth hormone/insulin-like growth factor 1) axis, which is still developing during the early postnatal period in mice. IGF-1 is highly sensitive to nutritional status [6] and actively involved in both fetal and postnatal growth. In addition, neuronal invalidation of the IGF-1 receptor (IGF-1R) recapitulates the phenotype of permanent postnatal growth delay observed in wild type mice underfed during lactation [7]. Permanent postnatal growth delays in these two mouse models are associated with altered development of the somatotropic axis due to pituitary hypoplasia of somatotrophs cells that produce GH.

Growth hormone releasing hormone (GHRH), a neuropeptide produced by neurons located in the arcuate nucleus of the hypothalamus (Arc), stimulates the secretion and transcription of GH by somatotrophs cells [8]. GHRH is also known to stimulate the proliferation and differentiation of somatotrophs progenitors during early postnatal life through the stimulation of the transcription factor Pit-1 [9]. Thus, underfed pups are characterized by specific delayed axonal growth in GHRH neurons, which require intact IGF-1 signaling [7, 10].

Other hormones that are highly sensitive to nutritional status may also be involved in the development of GHRH neurons. Among them, insulin is particularly relevant, because it is highly sensitive to nutritional status and has been shown to program metabolic disorders [11, 12]. As observed with IGF-1, insulin is secreted during the early postnatal period in response to amino acid, and its receptor is expressed by neurons at various levels in many nuclei of the brain. Insulin receptor is notably strongly expressed in the Arc nucleus [13]. However, neurons sub-populations expressing this receptor are still not characterized yet. Furthermore, insulin and IGF-1 share several structural and functional similarities, as well as downstream effectors [14–16]. However, whether insulin modulates axonal growth of Arc neurons during lactation in a nutrition-dependent context (normally fed *versus* underfed pups) is still unknown. We thus investigated the effect of insulin on the axonal development of the Arc GHRH neurons, using an *in vitro* model of Arc explants harvested from GHRH-eGFP pups. In addition to GHRH neurons, we completed our study with another functionally distinct neuronal population in order to highlight potential specificities. We choose the orexigenic Agouti-related peptide (AgRP) neurons. Indeed, we previously studied the impact of IGF-1 on these AgRP neurons [10]. Moreover, the sensitivity of this population to insulin have not been studied, in contrast to a third arcuate neuronal population, the POMC neurons [17]. Our results showed that insulin does not directly stimulate axon growth of developing Arc neurons *in vitro*. However, insulin may impact differently Arc neuronal subpopulations, which may also depend on nutritional conditions. Our results also showed that IGF-1-specific stimulation of axonal growth of GHRH neurons is not influenced by insulin signaling.

## Materials & methods

### Animal model

Arcuate explants were prepared, as previously described [10], from the GHRH-eGFP C57Bl/6J mouse line generated by I.C. Robinson *et al*. used by many laboratories [10, 18–22]. Mice were housed under standard SOPF conditions in individually ventilated cages at 22°C with a 12-h light/dark cycle (08:00 am– 08:00 pm) and free access to water and a commercial rodent diet (LASQCdiet Rod18-R, sterilized; LASVendi, Germany) (Table 1). Heterozygous GHRH-eGFP males (GHRH-eGFP^+/0^) maintained on a C57Bl/6J genetic background were bred with wild type C57Bl/6J females. Pregnant dams were isolated and housed individually to ensure that pup nutrition during lactation was provided by only one dam. Nutritional restriction during lactation was achieved for the entire litter by cross-fostering pups at birth: “normally fed” pups were obtained from a litter size of six pups per dam and “underfed” pups from a litter size of 10 pups per dam, as previously described [5, 10]. Male and female pups were homogenously balanced in each litter. For experimentation and statistical analyses, all pups originated from at least three different litters to avoid maternal bias. Animals were sacrificed by rapid decapitation. All animal experimentation was performed in accordance with institutional directives and French laws for the care of laboratory animals and approved by the French national ethics committee 05 Charles Darwin (Project protocol agreement number Ce5/2012/006).

**Table 1:** Composition of the LasQCdiet Rod18-R chow.

**Main nutrients**			**Amino acids per Kg**		
Protein	18.9	%	Arginine	9.0	g
Fat	5.3	%	Cysteine	4.0	g
Fibre	3.9	%	Histidine	4.5	g
Ash	7.0	%	Isoleucine	7.0	g
N-free-Extracts	53.1	%	Leucine	21.0	g
Dry matter	88.0	%	Lysine	9.0	g
			Methionine	4.5	g
Energy/kg			Phenylalanine	10.0	g
GE (gross)	16.6	MJ	Threonine	6.5	g
ME (metabolic	13.9	MJ	Tryptophan	2.0	g
			Tyrosine	7.0	g
**Minerals per kg**					
Calcium	10.0	g	**Vitamins per Kg**		
Phosphorus	6.5	g	Vit. A	15.000	I.E.
Sodium	3.0	g	Vit. D3	1.200	I.E.
Magnesium	2.5	g	Vit. E	90	mg
			Vit. K	5	mg
**Trace element per kg**			Thiamine (B1)	15	mg
Iron	200.0	mg	Riboflavin (B2)	10	mg
Iodine	4.0	mg	Pyridoxine (B6)	10	mg
Copper	15.0	mg	Cobalamin (B12)	50	mg
Cobalt	1.5	mg	Biotin	200	μg
Manganese	120.0	mg	Choline	1.000	mg
Selenium	0.2	mg	Folate	2	mg
Zinc	75.0	mg	Niacin	40	mg
			Pantothenate	20	mg
**Fatty acids per kg**					
C16 :0	7.5	g	C18 :1	13.0	g
C 18 :0	3.0	g	C18 :2	21.0	g
C20 :0	0.2	g	C18 :3	13.0	g

The LasQCdiet Rod18-R chow is provided ad libitum to mice and dedicated for gestation, lactation and growth of young animals. Ingredients are Wheat, wheat bran, corn gluten, wheat meal, corn, oats, barley, linseed oil, calcium carbonate, brewer's yeast, molasses, Vitamin-, minerals, vitamins/trace elements-mix, in descending order, respectively. It does not contain soya, fish, coating. The product is sterilized by a gamma-irradiation at minimum 21 kGy.

### Explant cultures

Explant cultures were prepared as previously described [10]. Briefly, brains were harvested from seven-day-old normally (total: 45 females and 45 males) or underfed (total: 19 females and 24 males) pups after rapid decapitation. For each experiment, brains were harvested from the entire litter and brains of both males and females were pooled before processing to obtain enough material from each litter to ensure high-quality explant cultures. Thus, although females are less sensitive to nutritional restriction than males [5], which may only mitigate the effects observed, arcuate nucleus explants were harvested from pups of both sexes to obtain a sufficient amount of material from each litter. Brain expression of eGFP was directly verified at the level of the median eminence by inverted fluorescence microscopy (Evos Cell Imaging, Evos). From the entire litter (6 pups for Normally fed and 10 pups for Underfed), brain were harvested and sliced at three hundred-μm thickness. Brain slices with Arc nucleus were kept apart and incubated on culture membranes (Whatmann) in MEM Medium (Life technologies) supplemented with 10% FCS, 0.5% glucose, and 1% penicillin/streptomycin, which include 2 slices/brain. Then the 2 Arcuate nuclei/ slice were micro-dissected from the brain and median eminence. Finally, each Arc nucleus was cut in 2 pieces. From normally fed pups, 48 (6 x 2 x 2 x 2) micro-dissected Arc explants were obtained and 36 of them were plated in one 4 wells’ plate, with 8 explants/well. From Underfed pups, three 4 wells’ plates were plated with 8 explants/well, and dedicated to different experiment to avoid any litter bias. Arc explants were uniformly plated in 4 well plate, representing the litter’s sex ratio. Arc explants were cultured in neurobasal medium (Life technologies) supplemented with methylcellulose, growth factors cocktail B27 supplement formulated either with insulin (B27 + Ins; ref 17504–044, Life Technologies) or without insulin (B27—Ins; ref A18956-01, Life Technologies), glucose, L-glutamine, and penicillin/streptomycin (Life technologies). B27 contains numerous hormones and growth factors to avoid cell-death of cultivated neurons. Its classic formulae (ref 17504–044) notably contains insulin, at a final concentration of 58 nM, according to our determinations. Cultures were performed with one control condition for each experiment. For each condition, the well was filled with eight explants. After 24 h in culture, the explants were treated with various concentrations of insulin (Actrapid, Novo Nordisk) and IGF-1 (R&D systems) alone or in combination with 10 μM picropodophyllotoxin (PPP, R&D) for another 24 h. The explants were then fixed for 30 min in 4% PFA at room temperature. After washes, explants were incubated for 30 min in 3% normal serum of goat or donkey, depending the secondary antibody used, plus 0.05% tween 20, and then incubated with primary antibodies directed against either neurofilament (ab72998, abcam; diluted at 1/1000), GFP (ab6556, abcam; diluted at 1/1000), AgRP (H-003-57, Phoenix pharmaceuticals, GmBH; diluted at 1/500) overnight at 4°C. After washes, explants were incubated for 2h at room temperature with the corresponding secondary antibodies (goat anti-chicken IgY (ab96949, abcam; diluted at 1/400), goat anti-rabbit IgG (ab96883, abcam; 1/400) and donkey anti-rabbit IgG (ab98499, abcam; diluted at 1/400) [10]. After washes, explants were mounted on slides with fluorescent mounting medium (Roti-mount fluorcare; Roth). All incubations were performed in 1x phosphate buffer saline (PBS)/ 1% normal serum/ 0.05% tween 20. Neurofilament and GFP were visualized together by dual- and AgRP by single-immunohistochemistry. Neurofilaments and AgRP were visualized under a 4X objective, and GFP under a 10X objective, as these axons were thinner, using an Olympus BX612 fluorescence microscope and DP71 CCD camera.

The analysis of axon length was performed with the NeuronJ plugin of ImageJ software as previously described [10, 23]. Briefly, the length of up to 40 axons/explants was measured individually. Then, the length was averaged for each explant and the global axonal length calculated as the mean of the eight quantified explants for each well. To account for variability of the explants cultures, the axonal growth (i.e. increase of axonal length in fold) were calculated for each treatment condition by normalization against the control (basal) condition. Each plate was considered to be one single experiment (n = 1) for statistical analyses. This experiment was reproduced 3–4 times independently for normally fed pups and 4–6 times for Underfed ones. Data are presented as the mean of axonal growth (in fold) calculated from various experiments depending on the test (see legends for the final n). The observed coefficient of error (OCE) between plates in Control and insulin 200nM stimulated conditions were 0.06 and 0.12 for NF neurons, 0.11 and 0.16 for GHRH-eGFP neurons and 0.20 and 0.22 for AgRP neurons, respectively.

Axons grow uniformly around the Arc explants on the ground of the culture dish that facilitate their individual length measurements. Indeed, *in vitro* neurons are not as tightly oriented as *in vivo*, which facilitate axons sprouting from explants. This is notably the case for GHRH neurons that are already slightly oriented even *in vivo* (see S1 Fig). Observation of GHRH neurons orientation *in vivo* has been studied by an *open book* approach. For the preparation of the *open book*, the medio-basal hypothalamus has been micro-dissected from the brain of 7 days-old male pups normally fed (n = 3). Arcuate nuclei of each hemisphere were open through the 3^rd^ ventricle while maintained to the median eminence (see Panel A in S1 Fig for protocol schema). These *open books* were fixed in PFA 4%, 20 min room temperature before to be subjected to an immunohistochemistry against GFP and neurofilament (NF) (see details above), and counterstained with DAPI. This approach allows the observation of the start of the axon, giving an idea of neurons orientation. Rostral direction was considered as 0°, dorsal direction as 90°, caudal direction as 180° and ventral direction as 270°. This experiment indicates that GHRH neurons are mainly dorsally oriented but equally go in rostral or caudal directions (see Panel B in S1 Fig).

### Biochemical analysis

Insulin concentration in B27 supplement was determined by ELISA (Access Ultrasensitive Insulin kit #33410 on an Access 2 analyzer, Beckman-Coulter), according to manufacturer instructions. The sensitivity was 0.03μU/mL, the intra-assay CV was 5.2–4.2% and the inter-assay CV was <10%.

### Statistical analysis

All data are presented as the mean ± SEM. Statistical analyses were performed with the GraphPad Prism 5 software. Body weight differences between normally-fed and underfed mice were analyzed by a t-test. Results for explant cultures were analyzed by two-way ANOVA with a Bonferroni correction for multiple comparisons. The precise molar concentrations-response effect within each group was further analyzed by one-way ANOVA with a Newman-Keuls multiple comparison test. Statistically non-significant results are abbreviated “NS” in the text. Statistically significant results are marked with asterisks, * *p* < 0.05; ** *p* < 0.01; and *** *p* < 0.001.

## Results

**Fig 1:**
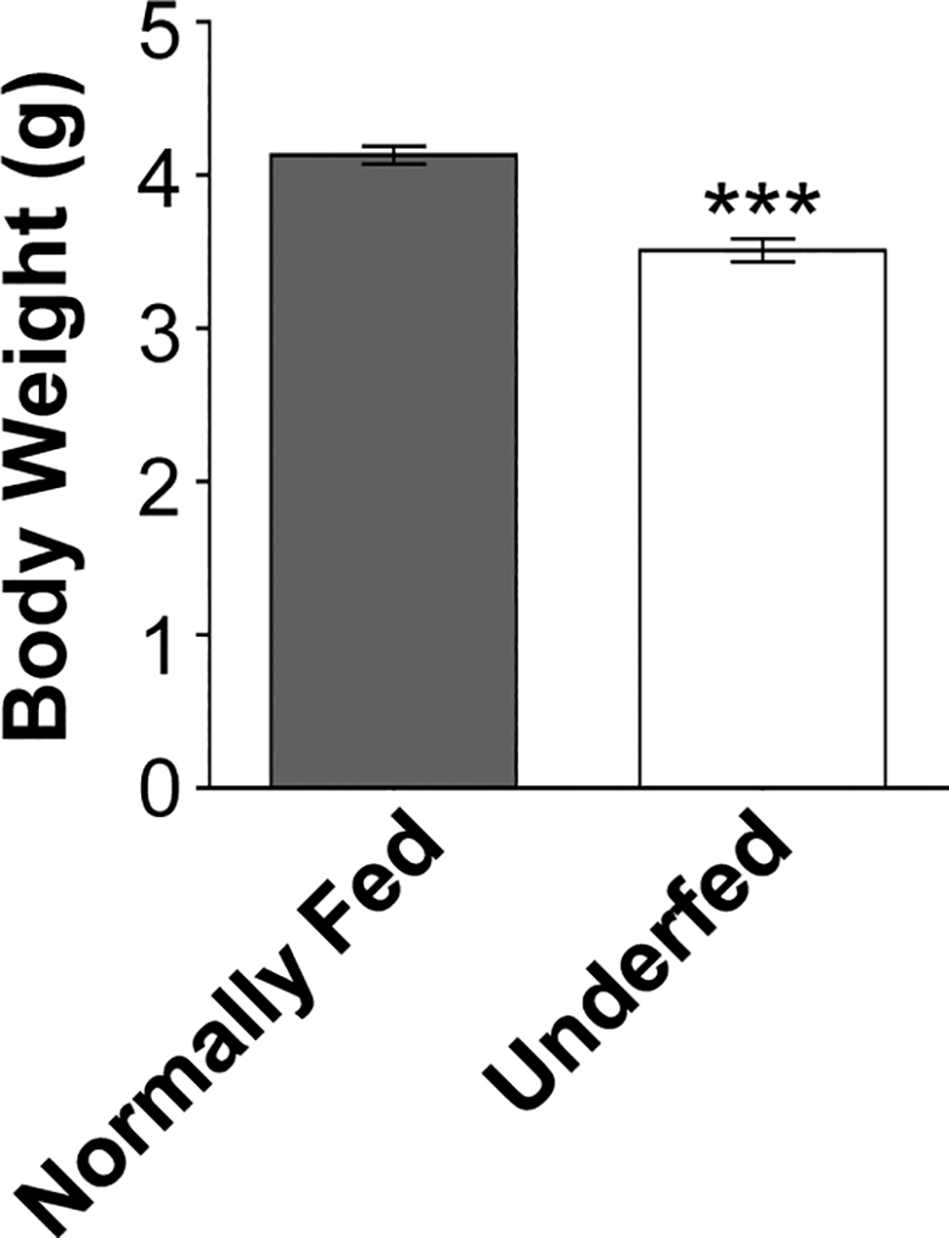
Underfed pups in larger litters have decreased insulin levels and are lighter. Increasing the litter size of lactating dams from six (normally fed) to 10 pups (underfed) lower the offspring body weight in 7 days-old pups of both sexes. Data are presented as the mean ± SEM, analyzed by the student t-test, *** *p* < 0.001.

Increasing litter size from six to 10 pups per dam delayed body weight gain in the larger litters relative to those with six pups. Underfed pups weighed 3.51 ± 0.07 g at seven days of age vs 4.13 ± 0.06 g for normally fed pups, both sexes included (Fig 1). We previously showed that underfeeding during lactation is associated with decreased circulating insulin levels of both sexes (underfed: 0.10 ± 0.03 ng/ml *vs* normally-fed: 0.15 ± 0.01 ng/ml, n = 7, *p* < 0.05) as well as glycaemia [5], consistent with other reports showing high sensitivity of insulin to nutritional status during the early postnatal period.

**Fig 2:**
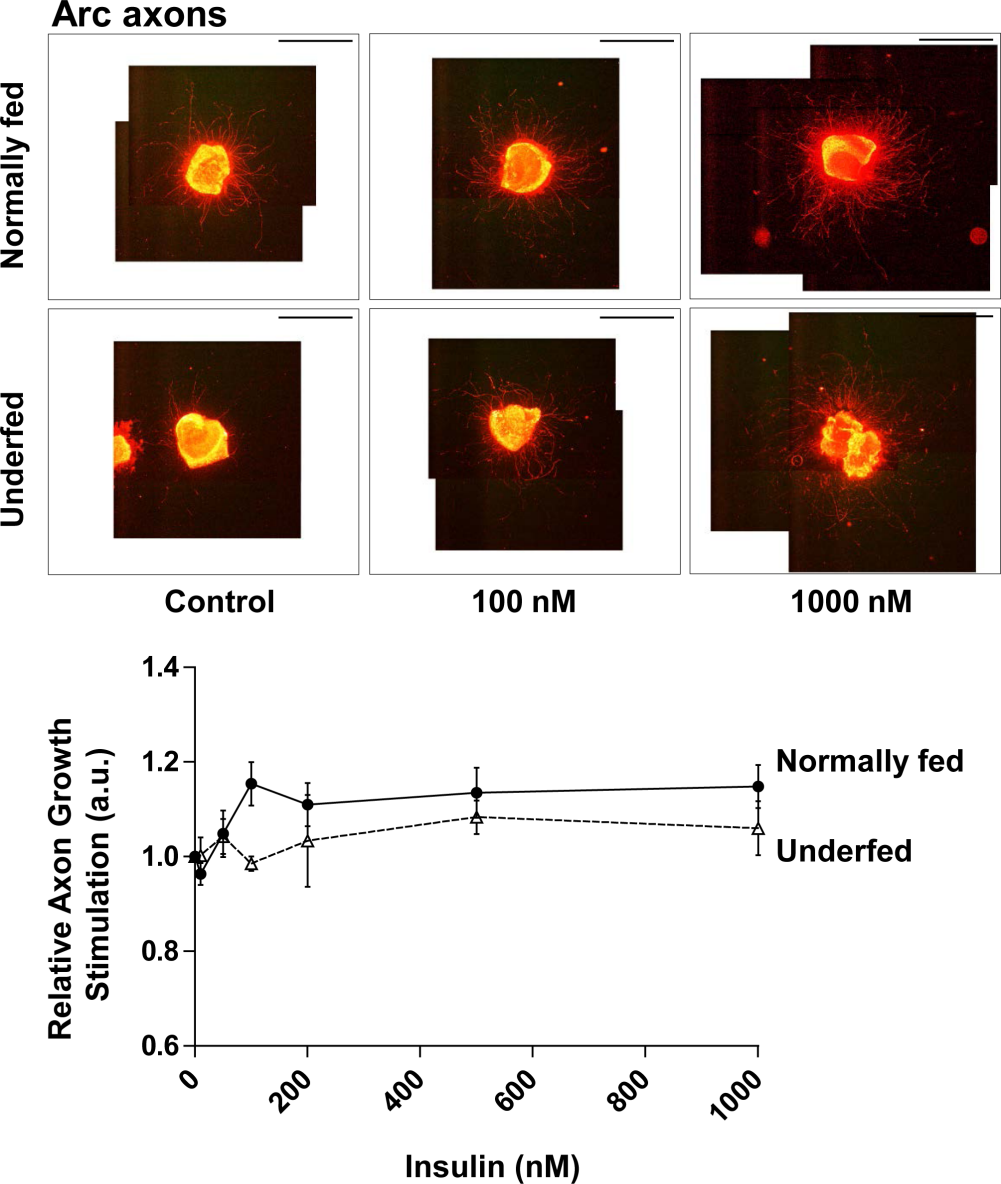
Response of arcuate neurons to insulin. Relative axonal growth in 24 h in whole arcuate neuron explants in the presence of various molar concentrations of insulin (0, 10, 50, 100, 200, 500 nM, and 1μM) was measured by immunohistochemistry against neurofilaments (NF). Arcuate explants were harvested from normally fed (dark circles and plain line) (n = 3–4) or underfed pups (open triangles and dotted line) (n = 6). Illustrative images of explants are presented above the graph for control (0 nM), 100 nM and 1000 nM of insulin. Scale bar indicates 2000 μm. Data are presented as the mean ± SEM. Results were analyzed by two-way ANOVA (see text) with a Bonferroni correction for multiple comparisons. The precise molar concentrations-response effect within each group was further analyzed by one-way ANOVA with a Newman-Keuls multiple comparison test. The results of all comparisons were non-significant.

We studied the effects of insulin on axonal growth of Arc neurons by cultivating arcuate nucleus explants in media supplemented with B27 without insulin. We first studied the axonal growth response of whole Arc neurons by neurofilament labelling. We also tested whether explants harvested from underfed pups showed altered sensitivity to insulin, as underfeeding may change the neuronal response to *in vitro* stimulation [10]. Globally, insulin presents an overall effect on axonal growth of neurons in Arc explant cultures (2-way ANOVA: *p* < 0.05) (Fig 2). In explants harvested from normally-fed pups, developing axons tended to be more numerous and grew more in the presence of insulin. Indeed, the number of analyzable NF^+^ axons tended to increase from 19 ± 7 to 26 ± 4 axons (NS) with increasing insulin concentration. In addition, there was a 1.15 ± 0.05-fold increase in axonal length when insulin was increased to 100 nM (Fig 2). However, these two tendencies did not reach statistical significance relative to the basal condition. Thus, insulin does not appear to significantly stimulate axonal growth in explants harvested from normally fed pups. Interestingly, explants harvested from one-week-old underfed pups appeared to be insensitive to insulin (2-way ANOVA: *p* = 0.050) (Fig 2). Notably, the length of axons was unchanged despite the increase of insulin concentrations (axonal growth: 0.99 ± 0.02-fold increase at 100 nM insulin vs Control).

**Fig 3:**
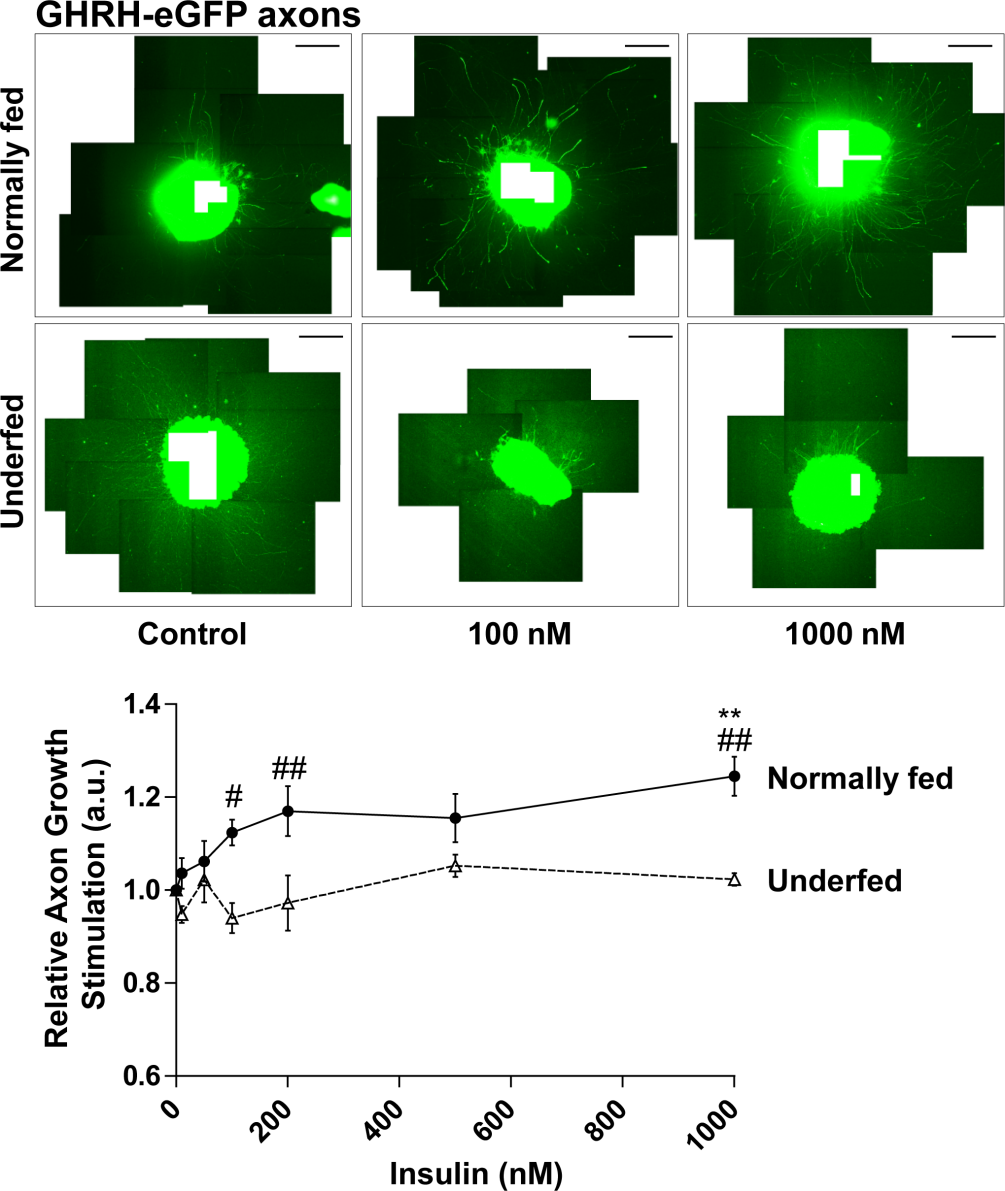
Response of GHRH neurons to insulin. Relative axonal growth in 24 h in the GHRH neuronal subpopulation of arcuate explants in the presence of various molar concentrations of insulin (0, 10, 50, 100, 200, 500 nM and 1μM) was measured. Arcuate explants were harvested from normally fed (plain circles and plain line) (n = 4) or underfed pups (open triangles and dotted line) (n = 6). Illustrative images of explants are presented above the graph for control (0 nM), 100 nM and 1000 nM of insulin. Scale bar indicates 600 μm. Pictures were reconstructed from multiple shots. The white rectangle in the centre of explants indicates the empty area. Data are presented as the mean ± SEM. Results were analyzed by two-way ANOVA with a Bonferroni correction for multiple comparisons (see text). Statistically significant results for comparisons of normally fed vs underfed pups are marked with hashtags, # *p* < 0.05; ## *p* < 0.01. The precise molar concentrations-response effect within each group was further analyzed by one-way ANOVA with a Newman-Keuls multiple comparison test. Statistically significant results relative to the control condition (0 nM) are marked with asterisks, ** *p* < 0.01.

Arc neuronal subpopulations respond differently to growth factors [10]. Thus, we specifically analyzed GHRH neurons. Globally, GHRH neurons appeared to be more sensitive to variations of insulin concentration (2-way ANOVA: *p* < 0.01) and nutritional status of the pups from which the explants originated (2-way ANOVA: *p* < 0.0001). The number of analyzable axons of GHRH neurons from explants harvested from normally-fed pups tended to be slightly higher in axons in the presence of insulin (from 23 ± 6 to 27 ± 4), without reaching statistical significance (NS). Axonal growth increased 1.12 ± 0.06-fold when insulin was increased to 100 nM (NS) (Fig 3). Here again, the global effect on axons length observed with increasing concentrations of insulin (two-way ANOVA), which could suggest a potential effect on GHRH neuronal welfare in Arc explants harvested from normally fed pups, does not appear to be associated with significant stimulation of axonal growth at physiological concentrations. The only statistically significant stimulation of axonal growth of GHRH neurons induced by insulin was observed at a very high concentration (1 μM) (*p* < 0.01), which is well-known to activate the IGF-1R [15]. GHRH neurons in Arc explants harvested from underfed pups were completely insensitive to insulin (axonal growth: 0.94 ± 0.04-fold induction at 100 nM insulin vs Control). Moreover, GHRH neurons from these cultures lost their capacity to respond to very high concentrations (1 μM) of insulin (Fig 3). This is consistent with our previous study [10], which showed that GHRH neurons in Arc explants harvested from underfed pups were resistant to the stimulation of axonal growth by IGF-1.

**Fig 4:**
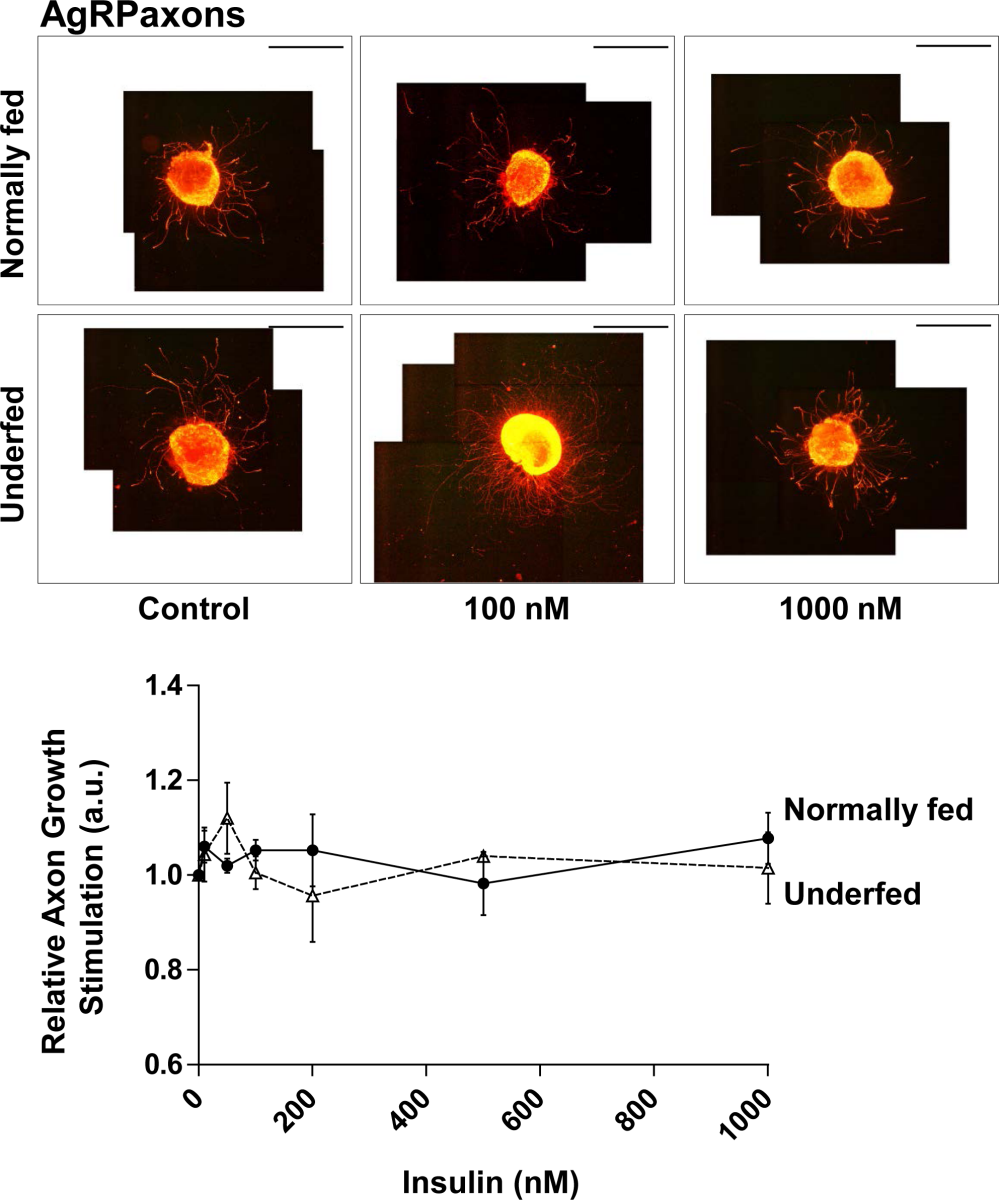
Response of AgRP neurons to insulin. Relative axonal growth in 24 h in the AgRP neuronal subpopulation of arcuate explants in the presence of various concentrations of insulin (0, 10, 50, 100, 200, 500 nM and 1μM) was measured. Arcuate explants were harvested from normally fed (plain circles and plain line) (n = 3) or underfed pups (open triangles and dotted line) (n = 4). Illustrative images are presented above the graph for control (0 nM), 100 nM and 1000 nM of insulin. Scale bar indicates 2000 μm. Data are presented as the mean ± SEM. Results were analyzed by two-way ANOVA (see text) with a Bonferroni correction for multiple comparisons. The precise molar concentrations-response effect within each group was further analyzed by one-way ANOVA with a Newman-Keuls multiple comparison test. The results of all comparisons were non-significant.

In parallel, we analyzed another neuronal sub-population of the Arc, orexigenic AgRP neurons. We focused on these neurons since they represent another major Arc neuronal subpopulation that we studied previously [10], and that the role of insulin on a third subpopulation (POMC neurons) has already been well studied [17]. AgRP neurons in explants harvested from normally-fed pups were insensitive to insulin, in contrast to the entire Arc neuronal population (NF^+^) or GHRH neurons. The number of analyzable axons was unchanged despite increasing the insulin concentration to 100 nM (22.9 ± 3.8 in Controls vs 18.5 ± 5.4 in AgRP neurons, NS). Axonal growth of AgRP neurons was also insensitive to insulin (1.05 ± 0.03-fold induction at 100 nM insulin vs Control, NS) (Fig 4). Moreover, AgRP neurons in Arc explants harvested from underfed pups showed the same insulin insensitivity (axonal growth: 1.01 ± 0.05-fold induction at 100 nM insulin vs Control, NS) (Fig 4). These data show that the AgRP neuronal sub-population is completely insensitive to insulin, irrespective of nutritional status.

In parallel, insulin may thus influence the regulation of axonal growth by growth factors, such as IGF-1. Indeed, insulin induces the activation of both the PI3K/AKT and the MEK/ERK signaling pathways, which are also activated by IGF-1. We previously showed that IGF-1 specifically stimulates axonal growth of GHRH neurons in Arc explants harvested from normally fed pups cultivated in a medium supplemented with B27 containing insulin (58 nM) [10]. We thus explored here whether the stimulatory effect of IGF-1 on GHRH neuron axonal growth could be influenced by the lack of insulin.

**Fig 5:**
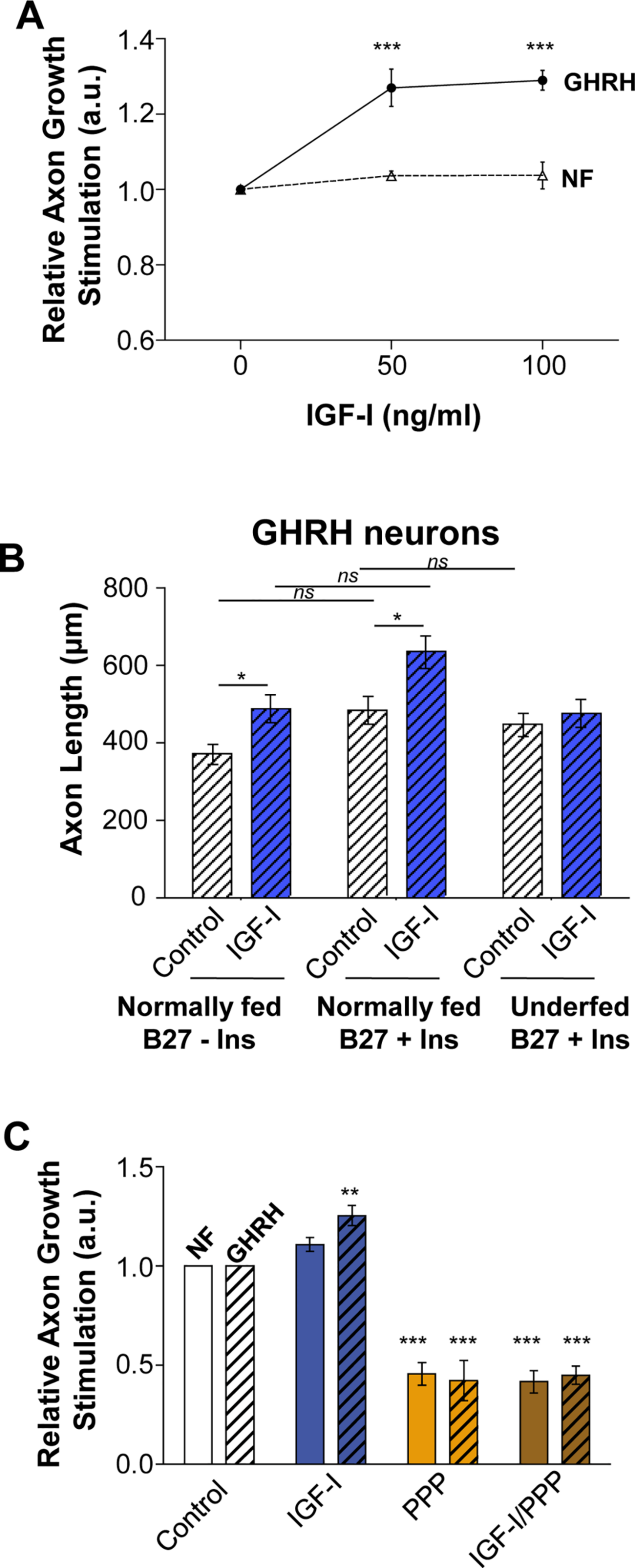
The specific axon-growth stimulatory effect of IGF-1 on GHRH neurons is not influenced by insulin. (**A**) Relative axonal growth in 24 h in the presence of various concentrations of IGF-1 (0, 50, 100 ng/ml) was measured for GHRH (closed circles and plain line) and whole-arcuate neurons (NF^+^; open triangles and dotted line) of arcuate explants harvested from normally-fed pups cultivated in media supplemented with B27 without insulin (n = 4). (**B**) Axon growth of GHRH neurons in Arc explants harvested from normally fed pups under Control and IGF-1-stimulatory conditions was determined in cultures supplemented with B27 with (B27 + Ins; n = 14–15) or without insulin (B27—Ins; n = 3–4). Axon growth of GHRH neurons in Arc explants harvested from underfed pups under Control and IGF-1-stimulatory conditions was determined in cultures supplemented with B27 with insulin (B27 + Ins; n = 9). (**C**) Axon growth in 24 h of NF^+^ (open bars) and GHRH (dashed bars) neurons was measured in response to IGF-1 stimulation (100 ng/ml) in arcuate explants harvested from normally-fed pups and cultivated in media supplemented with B27 + insulin, and involvement of the IGF-1R evaluated by treatment with the specific IGF-1R inhibitor picropodophyllotoxin (PPP) (n = 5–6). All data are presented as the mean ± SEM. The ANOVA analysis indicate a significant overall effect of the type of B27 formulae (p = 0.010) and also of IGF-I stimulation (p<0.05). However, post-hoc comparisons between B27+Ins vs B27-Ins with a Bonferroni correction for multiple comparisons do not reach statistical significance, either in Control condition or in IGF-I stimulated condition. Statistically significant results are marked with asterisks, X vs Control (0 ng/mL of IGF-I): *p < 0.05; **p < 0.01; ***p < 0.001.

We determined the axonal growth response of GHRH neurons to various concentrations of IGF-1 in Arc explants cultivated in a medium supplemented with B27 without insulin. As previously observed, only axonal growth of GHRH neurons were stimulated by IGF-1 (50 and 100 ng/ml) relative to control conditions, whereas whole Arc neurons (NF^+^) were insensitive (Fig 5A). These results suggest that insulin may not influence the response of GHRH to IGF-1 stimulation. We compared the behavior of GHRH neurons of Arc explants cultured under control and IGF-1-stimulatory conditions with or without insulin by comparing absolute axon length (μm), despite high variability between experiments. Increase of axon length of GHRH neurons was stimulated by IGF-1(IGF-1 vs Control: B27 + Ins = 1.32; B27—Ins = 1.23 fold), irrespective of the presence of insulin in the media (Fig 5B). In agreement with the insulin molar concentrations-response experiment described above, GHRH neurons of Arc explants cultured under both control and IGF-1 stimulatory conditions had longer axons when cultivated in media supplemented with B27 + insulin (Control B27+Ins: 514 ± 39 μm) than those cultivated in media supplemented with B27 without insulin (Control B7-Ins: 369 ± 15 μm). These results confirm a global positive effect of insulin on the welfare of GHRH neurons (2-way ANOVA: *p* < 0.05) (Fig 5B). The ANOVA analysis indicate a significant overall effect of the type of B27 formulae (p = 0.010) and also of IGF-I stimulation (p<0.05). However, post-hoc comparisons between B27+Ins vs B27-Ins do not reach statistical significance, either in Control condition or in IGF-I stimulated condition (Fig 5B). Arc explants harvested from Underfed pups present GHRH neurons with axonal length about 445 ± 33 μm in Control condition in a medium supplemented with the B27 formulae that contain insulin, and are not stimulated by addition of IGF-1, as previously published [10].

Finally, we also confirmed that IGF-1-induced axonal growth of GHRH neurons does not involve the insulin receptor and was strictly mediated through the activation of the IGF-1R. We cultivated explants of arcuate nucleus harvested from normally fed pups in a media supplemented with classic B27 that contains insulin and inhibited the IGF-1 stimulating effect using the highly specific IGF-1R inhibitor, picropodophyllotoxin (PPP). PPP completely abrogated the stimulation of axonal growth of GHRH neurons by IGF-1 (Fig 5C). These data suggest that the stimulation of GHRH neuron axonal growth by IGF-1 acts through stimulation of the IGF-1R.

## Discussion

This study was first designed to determine if insulin could program growth by stimulating the axonal growth of GHRH neurons. Insulin during the early post-natal period is already secreted in response to nutrition. However, Insulin secretion by first generation of beta-islets in young pups is not that sensitive to glucose levels and responds to amino acids [24]. The second generation of Beta islet cells, generated at the end of lactation, which are observed in adults are those who respond efficiently to glucose variations. Thus, insulin could have an impact on Arc neurons axonal growth, as it has previously shown for leptin and IGF-1 [10, 25]. Here, we show that insulin does not directly stimulate axonal growth nor influence IGF-1-dependent GHRH neuron axonal growth, despite activation of the same signaling pathways. In agreement with numerous reports on pro-survival effects of insulin on neuronal cultures *in vitro* [26–28], our data could also suggest a potential effect of insulin on welfare of neurons of the arcuate nucleus of the hypothalamus in a model of *in vitro* explant cultures. Our results also suggest differential sensitivity of Arc neuronal subpopulations, in which GHRH neurons are sensitive to insulin stimulation and AgRP neurons completely insensitive. Moreover, the nutritional environment during neuronal development also appears to influence neuronal sensitivity to insulin *in vitro* (see S1 Table for summary).

Indeed, increasing insulin concentration in Arc explants cultures is associated with a global effect as indicated with the significant two-way ANOVA analysis. Axons from Arc explants were slightly longer and more numerous in the presence of insulin even at low concentrations (~50 nM). This is consistent with numerous reports that have led to the use of insulin in almost all supplements dedicated to neuronal cultures, such as B27 [26–28]. However, axon length increase does not reach statistical significance, indicating that insulin does not directly stimulate axon growth at physiological concentration. The slight increase of neurons length and number may be an indirect consequence of pro-survival effects of insulin. This is also strongly suggested by the increase of axon length measured when Arc explants are cultivated in medium supplemented with the B27 formulae that contain insulin (Fig 5B). However, since none of these effects reach the statistical significance, it is difficult to conclude. Since these experiments were performed on Arc explants that can be cultivated for 48h only, this lets not enough time for neurons stabilization after micro-dissection and study the proper effect of insulin on neurons survival. It is thus delicate to directly observe the effects of insulin on neurons survival. Furthermore, Arc explants show a strong background staining after IHC, avoiding the study of cell body or nucleus of neurons.

Our data concerning the development of Arc neurons in a nutritionally restricted environment ask the question about a potential change in their susceptibility to anti-apoptotic effects of insulin. Indeed, NF^+^ and GHRH neurons appeared to be less sensitive to the effects of insulin when harvested from underfed pups, regardless of the concentration.

A similar decrease in sensitivity has been previously reported for the stimulation of axonal growth of GHRH neurons by IGF-1. Indeed, the specific effect of IGF-1 on axonal growth of GHRH neurons observed in Arc explants harvested from normally fed pups was completely abolished in explants harvested from underfed pups [10]. This was associated with the specific loss of IGF-1-induced AKT phosphorylation, whereas the ERK/MEK signaling pathway was still fully responsive in the same explants [10]. IGF-1 and insulin share both PI3K/AKT and ERK/MEK signaling pathways. Indeed, the specific implication of AKT in insulin-induced survival of neuronal cultures has been suggested [27]. Unfortunately, it has been difficult to directly test this hypothesis because insulin does not significantly stimulate axonal growth of GHRH neurons in our model. Indeed, the addition of insulin at physiological concentrations to Arc explant cultures was not associated with significant stimulation of axonal growth of GHRH neurons relative to the control condition. The only significant increase of axonal growth observed was for GHRH neurons of explants harvested from normally fed pups stimulated with a very high concentration (1μM) of insulin, which is known to activate the IGF-1R.

These results suggest that insulin is not directly involved in the stimulation of axonal growth. However, the stimulation of axonal growth of GHRH neurons by very high concentrations of insulin confirm the specific involvement of the IGF-1R [15], as previously observed [10]. Thus, the stimulation of axonal growth appears to be specifically induced by IGF-1, even though insulin and IGF-1 share common signaling pathways. Furthermore, comparison of the behavior of GHRH neurons cultivated in medium supplemented with B27, with or without insulin, showed that IGF-1-induced stimulation of their axonal growth is not influenced by insulin as the relative axonal growth induced by IGF-I is similar in both conditions. Finally, we also confirmed that the stimulatory effect of IGF-1 on GHRH axonal growth specifically involves the IGF-1R, as it was fully abrogated by co-incubation with PPP, which is highly specific for the IGF-1R and does not act on the insulin receptor [29, 30].

The impact if insulin on axonal growth of Arc neurons was measured *in vitro* on arcuate explants cultures, micro-dissected from 7 day-old GHRH-eGFP pups. Indeed, GHRH neurons are still developing during the early postnatal period and were previously demonstrated to respond to IGF-I stimulation *in vitro* [5, 7, 10]. Hypothalamic neurons grow up to around P16 [10, 25, 31]. Thus, stimulation of axon growth should be studied during the development of neurons. Regarding the Arc explants cultures, P7 was determined as the best age for axon re-growth *in vitro* after a micro-dissection (L.K. & E.M., personal communication) for GHRH neurons [10], which is in agreement with other studies on AgRP and POMC neurons [25]. This Arc explants culture approach has been used by many groups to study axon growth and is reliable as it present observed coefficient of error (OCE) globally below 20% between experiments (see methods section for details). As illustrated in picture Figs 2–4, axons grow uniformly around the Arc explants on the ground of the culture dish that facilitates their individual length measurements. Indeed, neurons *in vitro* are as not tightly oriented as *in vivo*, which facilitate axons sprouting from explants. This is notably the case for GHRH neurons that are already slightly oriented even *in vivo* (S1 Fig).

However, we cannot eliminate a potential effect of sex in this study. Indeed, since a minimum amount of explants is required for the quality of explants cultures, brain from both males and females were pooled before to be processed all together. Thus, although females are less sensitive to nutritional restriction than males [5], which may dampen the effects observed, arcuate nucleus explants were harvested from pups of both sexes to obtain a sufficient amount of material from each litter. In addition, one prior study has shown that they are sex and age differences in GHRH neuron development [22]. Indeed, it was reported that GHRH neurons increase in both sexes during the P1-P10 period, males having more GHRH neurons than females. After P20, the number of GHRH neurons stabilized in males, and continued to increase in females [22], but our data does not indicate if Arc neurons of P7 male and female pups react differently.

The AgRP neuronal sub-population of the Arc was insensitive to the beneficial effect of insulin. The axonal length of AgRP neurons was unaltered by insulin exposure, regardless of the nutritional status of the pups from which they were harvested, in contrast to NF^+^ and GHRH neurons. These data suggest that all sub-population of Arc neurons do not have the same susceptibility to the trophic support offered by insulin. Indeed, differential insulin sensitivity depending on the origin and sub-type of neurons has been previously observed [27, 28]. Prolonged exposure of human neural stem cells, as well as differentiated human neuron cultures, to insulin concentrations optimized for those of rodents is toxic. Human neural stem cell cultures are extremely sensitive to insulin, unlike those of rodents, and evidence shows that human neuronal cell death in culture involves the development of insulin resistance [27]. Moreover, Singh *et al*. reported differential insulin sensitivity in rat neuron cultures, depending on their origin [28]. They showed that sensory neurons derived from rat dorsal root ganglia exposed to μM concentrations of insulin developed resistance, whereas those originating from the central nervous system did not. Our results suggest that orexigenic AgRP neurons, which are strongly exposed to peripheral hormones, are also less sensitive to insulin than GHRH neurons, although they are both located in the same hypothalamic nucleus. We limited our study to AgRP neurons because the effect of insulin on POMC neurons, the other arcuate neuronal sub-population involved in the control of food intake, has been well-studied by Vogt *et al*. [17]. They showed that alterations of the innervation of target nuclei by POMC neurons in offspring of lactating dams fed a high fat diet are modulated by insulin signaling. The specific deletion of the insulin receptor in POMC neurons rescued their innervation in the posterior paraventricular nucleus. This effect appeared to be specific as the anterior paraventricular nucleus still showed less POMC innervation and innervation by AgRP neurons of both the anterior and posterior paraventricular nucleus was not rescued [17]. These results show that a portion of POMC neurons is sensitive to insulin signaling. The apparent specificity of neuron sub-populations, as well as of their innervation target, to nutritional hormones, such as insulin, is yet to be elucidated. Although insulin is tightly controlled by nutrition in the early postnatal period, it may not be involved in the programming of the somatotropic axis and linear growth. Extensive analyses will be required to understand this system, of which the high complexity may be a key element for the fine tuning and programming of life trajectories.

## Supporting information

S1 Table Summary of impacts of insulin on primary arcuate neurons culture. Statistical analyses are summarized for impacts of insulin and/or IGF-1 on axonal growth of different neuronal sub-population in Arc explants harvested from normally fed or underfed pups. 2 way ANOVA were performed with Bonferonni as multiple comparison post hoc test, 1 way ANOVA was performed with a Newman-Keuls post hoc test.
(PDF)

S1 Fig Orientation of GHRH neurons in the Arc nucleus. **A**, For the preparation of the *open book*, the medio-basal hypothalamus has been micro-dissected from the brain of 7 days-old GHRH-eGFP male pups normally fed (n = 3). Arcuate nuclei of each hemisphere were open through the 3^rd^ ventricle while maintained to the median eminence. These *open books* were fixed and subjected to an immunohistochemistry against GFP (green) and neurofilament (NF) and counterstained with DAPI. This approach allows the observation of the start of the axon, giving an idea of neurons orientation. **B**, Rostral direction was considered as 0°, dorsal direction as 90°, caudal direction as 180° and ventral direction as 270°. Individual axons analyzed are depict with thin lines and means with thick ones. The number of axons observed in each quadrant is indicated around.
(PDF)
