# Supplementary material for: Impact of insulin on primary arcuate neurons culture is dependent on early-postnatal nutritional status and neuronal subpopulation
Source: PLoS One. 2018 Feb 21;13(2):e0193196. doi: 10.1371/journal.pone.0193196 (PMC5821369; doi:10.1371/journal.pone.0193196)
Supplement: S1 Table — Statistical analyses are summarized for impacts of insulin and/or IGF-1 on axonal growth of different neuronal sub-population in Arc explants harvested from normally fed or underfed pups. 2 way ANOVA were performed with Bonferonni as multiple comparison post hoc test, 1 way ANOVA was performed with a Newman-Keuls post hoc test. (PDF) [file pone.0193196.s001.pdf]

S1 Table: Summary

Parameters	Test		Comparison	p value
Body Weight		t-test	Normally fed vs Underfed	p<0.0001
Axon growth of NF neurons in response to Insulin	2 way ANOVA		Concentration Insulin	p<0.05
			Nutrition effect	ns
			Interaction	ns
		Bonferonni	Normally fed vs Underfed	ns
	1 way ANOVA	Newman Keuls	Insulin concentrations vs Control	ns
Axon growth of GHRH neurons in response to Insulin	2 way ANOVA		Concentration Insulin	p<0.001
			Nutrition effect	p<0.0001
			Interaction	p<0.05
		Bonferonni	Normally fed vs Underfed	100 nM p<0.05
				200 nM p<0.01
				1000 nM p<0.001
	1 way ANOVA	Newman Keuls	0 vs 1000nM	p<0.01
Axon growth of AgRP neurons in response to Insulin	2 way ANOVA		Concentration Insulin	ns
			Nutrition effect	ns
			Interaction	ns
	1 way ANOVA	Newman Keuls	Insulin concentrations vs Control	ns
NF and GHRH axonal growth response to IGF-1 in medium supplemented in B27-Ins	2 way ANOVA		IGF-I concentration	p<0.0001
			Neurons type	p<0.0001
			Interaction	p<0.0001
		Bonferonni	NF vs GHRH	50 nM p<0.0001
				100 nM p<0.0001
GHRH Axon growth response to IGF-I stimulation depending of the medium supplementation and nutritional status	2 way ANOVA		IGF-I stimulation	p<0.05
			B27 formulae	p=0.01
			Interaction	ns
		Bonferonni	Control vs IGF-I	normally fed p<0.05
				Underfed ns
			B27 formulae	ns
NF and GHRH axonal growth response to IGF-I and PPP	2 way ANOVA		Treatment	p<0.0001
			neurons type	ns
		Bonferonni	Control vs IGF-I	NF ns
				GHRH p<0.01
			Control vs PPP	NF p<0.0001
				GHRH p<0.0001
			Control vs IGF/PPP	NF p<0.0001
				GHRH p<0.0001
