# Supplementary figures and images for: Impact of insulin on primary arcuate neurons culture is dependent on early-postnatal nutritional status and neuronal subpopulation
Source: PLoS One. 2018 Feb 21;13(2):e0193196. doi: 10.1371/journal.pone.0193196 (PMC5821369; doi:10.1371/journal.pone.0193196)

**A**

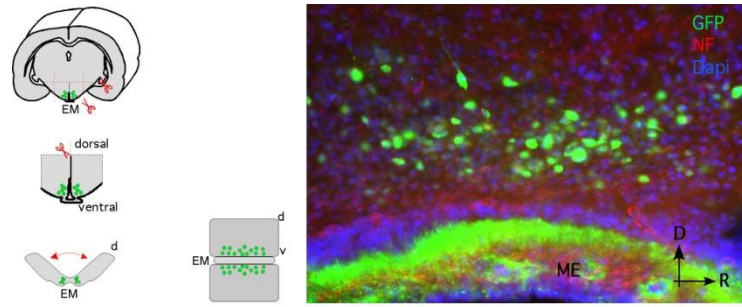

**B**

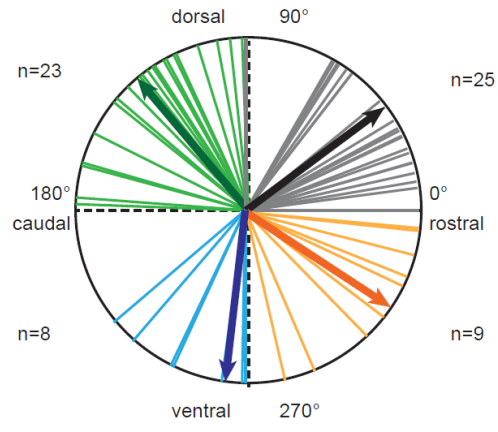

**S1 FIGURE**

Supplement: S1 Fig — A, For the preparation of the open book, the medio-basal hypothalamus has been micro-dissected from the brain of 7 days-old GHRH-eGFP male pups normally fed (n = 3). Arcuate nuclei of each hemisphere were open through the 3rd ventricle while maintained to the median eminence. These open books were fixed and subjected to an immunohistochemistry against GFP (green) and neurofilament (NF) and counterstained with DAPI. This approach allows the observation of the start of the axon, giving an idea of neurons orientation. B, Rostral direction was considered as 0°, dorsal direction as 90°, caudal direction as 180° and ventral direction as 270°. Individual axons analyzed are depict with thin lines and means with thick ones. The number of axons observed in each quadrant is indicated around. (PDF) [file pone.0193196.s002.pdf]
